# A birch ELONGATED HYPOCOTYL 5 gene enhances UV-B and drought tolerance

**DOI:** 10.48130/forres-0024-0019

**Published:** 2024-06-19

**Authors:** Shangzhu Gao, Xiaohui Chen, Meihan Lin, Yibo Yin, Xiaoyi Li, Yaguang Zhan, Ying Xin, Fansuo Zeng

**Affiliations:** 1 State Key Laboratory of Tree Genetics and Breeding, Northeast Forestry University, Harbin 150040, China; 2 College of Life Science, Northeast Forestry University, Harbin 150040, China; 3 School of Forestry, Northeast Forestry University, Harbin 150040, China

**Keywords:** ABA, *Betula platyphylla*, *BpHY5*, bZIP, Drought stress, UV-B

## Abstract

UV-B radiation and drought majorly restrict plant growth, particularly in summer. ELONGATED HYPOCOTYL 5 (HY5), a bZIP transcription factor (TF), has a beneficial impact on photomorphogenesis. However, the sequence of *HY5* from *Betula platyphylla* (*BpHY5*) has not been identified and the gene functions remain unclarified. We cloned the sequence of BpHY5, which was targeted to the nucleus. The hypocotyl phenotypes of heterologous expression in *Arabidopsis thaliana* and reverse mutation showed that *BpHY5* is homologous to *AtHY5*. The expression of *BpHY5* was increased in response to UV-B radiation, drought conditions, and the presence of abscisic acid (ABA). The overexpression of *BpHY5* resulted in increased tolerance to UV-B radiation and drought and decreased ABA sensitivity with higher germination and greening rate, more developmental root system, stronger reactive oxygen species scavenging ability, and lower damage degree. The lignin content under UV-B condition of *BpHY5*/Col was higher than that of Col. Furthermore, overexpressing *BpHY5* up-regulated the expression of genes related to tolerance (*NCED3/9*, *ABI5*, *DREB2A*, *RD20*, *ERF4*, *NDB2,* and *APX2*). In brief, the study suggests that *BpHY5* from birch serves as a beneficial modulator of plant responses to UV-B radiation and drought stress.

## Introduction

The UV-B light in sunlight (280−315 nm) plays a crucial role in the growth, development, and adaptation of plants. UV-B has been regarded as a potential stressor for organisms, as mild UV-B induces plant adaptive responses, while severe UV-B causes plant metabolic disturbances, such as causing damage to DNA, protein, photosynthetic machinery, photosynthetic processes, and arresting the cell cycle^[1]^. UV-B signals cause UV RESISTANCE LOCUS 8 (UVR8) homodimers to split into monomers, which then interact with CONSTITUTIVELY PHOTOMORPHOGENIC 1 (COP1), and the E3 ubiquitin of COP1 is regulated by SUPPRESSOR of PHYA (SPAs)^[2]^. COP1/SPA regulates the activity of photomorphogenesis transcription factors (TFs), such as ELONGATED HYPOCOTYL 5 (HY5), HY5-HOMOLOG (HYH), and some B-box TFs^[3]^. HY5, a bZIP TF, is a positive regulator of photomorphogenesis. Under all light conditions, *hy5* mutants of *Arabidopsis* display elongated hypocotyl^[4]^. Besides, HY5 is also involved in regulating stress responses. There is evidence that nearly one-third of *Arabidopsis* gene promoters are bound to HY5^[5]^. *HY5* increases plants' survivability and promotes anthocyanin biosynthesis under UV-B stress by negatively regulating DECREASE WAX BIOSYNTHESIS (DEWAX) expression^[6]^. HY5 binds to the promoter of *ABA-INSENSITIVE 5* (*ABI5*) with high affinity, which can be significantly enhanced by ABA^[7]^.

As major environmental factors, water deficiencies limit the growth, development, establishment, and productivity of forest stands. Lack of water causes osmotic stresses, affects plants growth and development, even leads to death. It is thought that TFs that recognize and bind to cis-acting elements of stress-responsive gene promoters may regulate drought tolerance. The basic leucine zipper (bZIP) is one of the largest and most conserved transcription factors in plants. The bZIP mainly contains two functional regions, a strictly conservative basic N-terminal DNA-binding region (N- × 7-R/K) with 18 amino acids and a leucine zipper dimerization domain. This aspect of the research suggests that bZIP TFs participate in various plant processes such as seed maturation, growth, light signaling, secondary metabolites biosynthesis, and stress response.

Abiotic stresses, such as UV-B and drought, can trigger both abscisic acid (ABA)-dependent and ABA-independent transcriptional responses in plants^[8]^. ABA is recognized as an essential hormone in plant development and stress responses. Endogenous ABA level is accumulated in response to water stress, inducing the expression of stress-related genes, such as dehydration-responsive genes. The exogenous ABA is added to model dehydration reactions, which induces the expression level change of various dehydration-responsive genes. Diversiform regulatory mechanisms of plant tolerance to abiotic mediated by bZIP TFs are related to the ABA signal. ABRE binding protein 9 (ABP9) binds to ABA-response element ABRE2 and enhances osmotic and oxidative stress resistances in maize^[9]^. Furthermore, *OsbZIP23*, a key regulator in ABA signaling, enhances drought tolerance and decreases the sensitivity of ABA in rice, positively regulating the expression of *OsPP2C49*^[10]^.

*Betula platyphylla* (birch) is a deciduous tree species with ecological and economic importance in the Northern Hemisphere, which is widely used in lumber, furniture, buildings, landscape trees, papermaking, medication, and cosmetics. Birch is regarded as a pioneer tree and is often found in open habitats (ridges, rocks, and deforested areas), resulting in increased susceptibility to UV-B and drought stress. On this basis, the present study proposes to explore the potential functions of *BpHY5* from birch, particularly for tolerance to UV-B and drought stresses. The research on the characteristics of *BpHY5* will be beneficial to genetic improvement, sustainable afforestation, and wood production of birch.

## Materials and methods

### Plant materials and growth conditions

The experiment was conducted at Northeast Forestry University, China (45.7203° N, 126.6346° E). The birch seeds were sterilized and cultured in Woody Plant Medium (WPM). After 1.5 months of growth, the seedlings were transplanted into the culture soil in the controlled environment under long-day (16 h light/8 h dark) conditions at 25 ± 2 °C and 60% relative humidity.

### Gene cloning and qRT-PCR analysis

Specific primer pairs were designed based on the sequence of transcription factor HY5 in birch (KJ466369) from NCBI (Supplemental Table S1). The full-length coding sequence of *BpHY5* was amplified by PCR using cDNA from wild-type birch as a template. Total RNA extraction and qRT-PCR were performed as previously described by Gao et al.^[11]^. The heatmaps were developed by TBtools.

### Bioinformatics analyses of *BpHY5*

The homologous HY5 proteins of *Arabidopsis thaliana*, walnut (*Juglans regia*), poplar (*Populus trichocarpa*), jujube (*Ziziphus jujuba*), *Cannabis sativa*, melon (*Cucumis melo*), cork oak (*Quercus suber*), peach (*Prunus persica*), castor bean (*Ricinus communis*), *Jatropha curcas*, *Eucalyptus grandis*, grape (*Vitis riparia*), and cotton (*Gossypium hirsutum*) were obtained by BLAST searches of the NCBI database. Phylogenetic trees were created using the Jones-Taylor-Thornton (JTT) model and neighbor-joining (NJ) method through MEGA-X. The conserved functional domains of HY5 were defined using TBtools. The conserved motif analysis was done by MEME and TBtools. SWISS-MODEL was used to predict tertiary structure. The promoter sequence of *BpHY5* was defined by the genome database of *Betula platyphylla* (https://phytozome-next.jgi.doe.gov/info/Bplatyphylla_v1_1) and BioEdit software. The promoter was predicted and analyzed by PlantCARE.

### Vector construction and subcellular location of *BpHY5*

The recombinant vector pROKII-BpHY5-GFP was introduced into *Agrobacterium* *tumefaciens* strain GV3101 cells to transform birch and *Arabidopsis*. The recombinant plasmid (pEarleyGate 103-35S::BpHY5-GFP) and empty vector (pEarleyGate 103-35S::GFP) were introduced into GV3101, which were transformed into onion epidermal cells, respectively. After 24 h of incubation, confocal microscopy LSM800 (Zeiss, Oberkochen, Germany) was used to observe GFP and DAPI.

### Transient transformation and stress treatment of birch seedlings

To analyze the time-specific expression of *BpHY5*, the samples of different periods (0, 3, 6, 9, 12, 15, 18, 21 h) from one-month-old birch in soil were collected. One-month-old birch in soil was treated with 0.6 W/m^2^ UV-B to analyze the expression pattern of *BpHY5* under different durations (0, 0.5, 3, and 6 h). The birch seedlings of 1.5-month-old were subjected to a 3, 6, or 12-h treatment with 200 mM mannitol and 10 μM ABA in medium to analyze the expression pattern under different abiotic stresses.

The method for transient transformation of birch seedlings was carried out according to Wang et al.^[12]^. The transgenic seedlings were subjected to a 3-h treatment with 200 mM mannitol and 10 μM ABA to determine physiological parameters and analyze the gene expression. To ensure accuracy, all samples were harvested in at least three biological replicates.

### Transformation and stress treatment of *Arabidopsis*

The mutants *hy5* (CS71, hy5-1) were from AraShare. The wild type used in the study was the Columbia line (Col). Transformation of *Arabidopsis* was performed using the floral dip method. The Homozygous T3 strain was obtained by self-crossing to be used in the experiment and the transgenic line exhibiting the highest level of *BpHY5* expression across independent lines and different genetic backgrounds was selected for further analysis. The seedlings of *Arabidopsis* (Col, *hy5*, *BpHY5*/*hy5,* and *BpHY5*/Col) in the medium were used to measure the hypocotyl length under darkness or visible light for 7 d.

For the UV-B tolerance assay, seed (Col, *hy5,* and *BpHY5*/Col) germination and greening rates were measured under varying UV-B levels (0.8 and 1.6 W/m^2^) from day 0 to day 6. The fresh weight and root length of 14-day-old seedlings were measured. Seedlings were transferred to soil at 7 d and grown for 7 d. Two-week-old plants were subjected to 0.8 and 1.6 W/m^2^ UV-B treatment for 10 d for measuring fresh weight, chlorophyll fluorescence, malondialdehyde (MDA), H_2_O_2_, superoxide anion content relative conductivity, and component content of stems.

For drought and ABA tolerance assay, seeds (Col and *BpHY5*/Col) were planted in a medium containing mannitol (100, 200 mM) and ABA (0.5, 1 μM) to measure germination and greening rates from day 0 to day 6.5. The 16-day-old seedlings were harvested to measure fresh weight, root length, MDA, superoxide anion content, and relative conductivity. The whole 16-day-old plants (Col and *BpHY5*/Col) were harvested to analyze the expression of tolerance-related genes. After being transferred to soil for 20 d, the 27-day-old plants were subjected to drought stress without water for 7 d and re-watered for 3 d.

### Chlorophyll fluorescence and staining

The chlorophyll fluorescence of *Arabidopsis* under UV-B was measured by kinetic chlorophyll fluorescence imaging systems (Heinz Walz GmbH, Free State of Bavaria, Germany). Fv/Fm = (Fm−Fo)/Fm. The leaves of materials were stained with DAB (1 mg/mL) and Evans blue (0.5%) overnight, and NBT (1 mg/mL) for 4 to 6 h, respectively.

### Physiological, biochemical measurements and cell wall component analysis

The relative conductivity was carried out as per the method described by Li et al.^[13]^. Water loss was analyzed with reference to the method described by Chen et al.^[14]^. The MDA, hydrogen peroxide, superoxide anion, lignin, cellulose, and hemicellulose content were quantified using a detection kit (Grace Biotechnology, Suzhou, China).

### Statistical analysis

Data presented as mean ± SD was analyzed using IBM SPSS 22 for statistical significance at *p* < 0.05. All data were analyzed in triplicate.

## Results

### Sequence alignment and phylogenetic analysis

**Figure 1:**
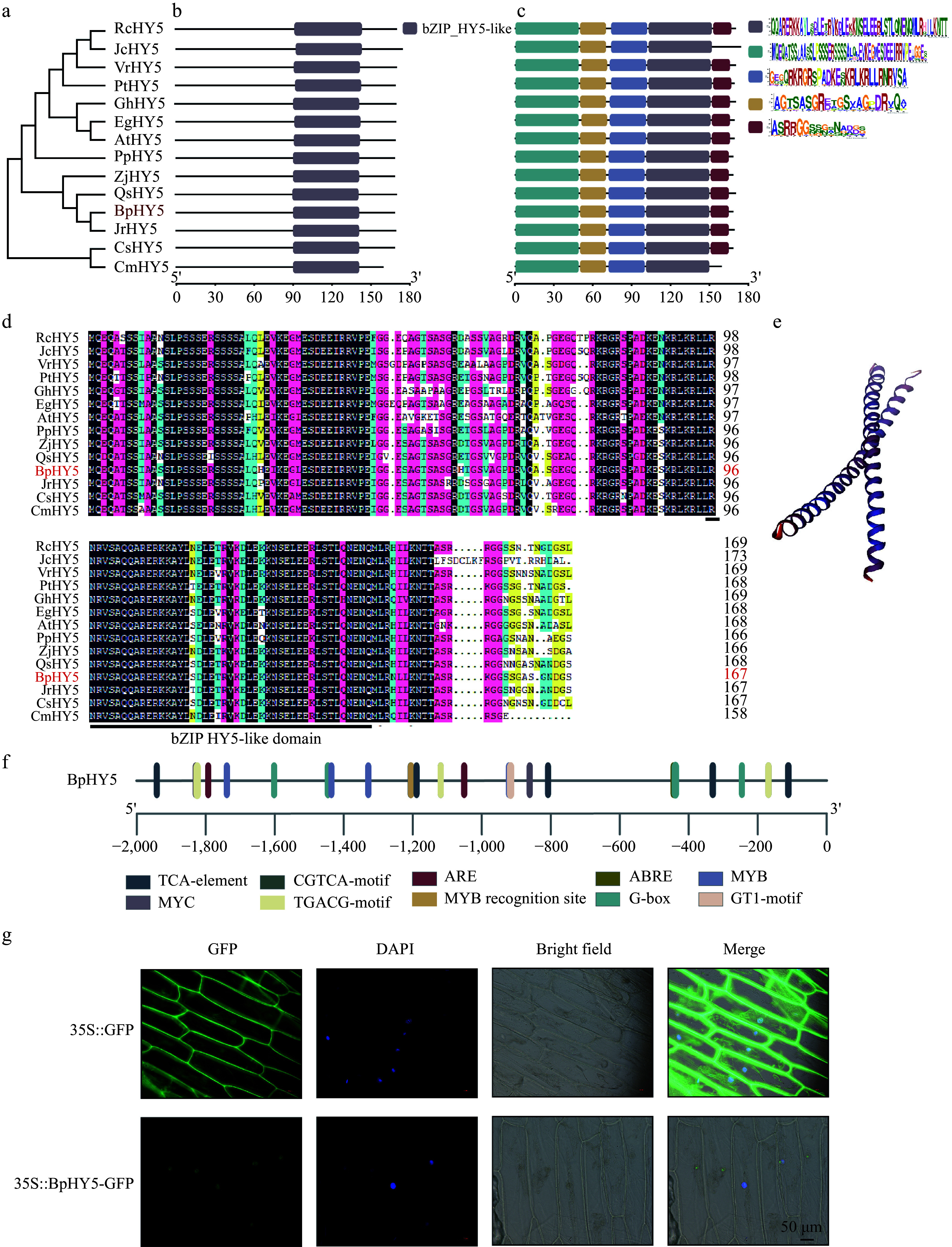
Phylogenetic, sequence analysis, and subcellular localization of BpHY5. (a) Phylogenetic tree of HY5. Predicted conserved (b) domain and (c) motifs of HY5 proteins, with five colored boxes representing five motifs. (d) Multiple sequence alignment. (e) The predicting 3D structure of BpHY5. (f) Cis-acting element analysis of the promoter sequence of *BpHY5*. (g) Subcellular localization of 35S::GFP and 35S::BpHY5-GFP. Images represent GFP, DAPI, bright field, and their merged, respectively. The scale bar represents 50 μm.

The full-length ORF of *BpHY5* (KJ466369) is 504 bp, encoding a putative protein of 167 amino acids. The phylogenetic analysis of BpHY5 and HY5s which were identified from the other thirteen plant species was performed (Fig. 1a). Amino acid sequence alignment indicated that BpHY5 contained bZIP_HY5-like conserved domain, that was similar to HY5s from other thirteen species, sharing 82.25%, 76.92%, and 73.81% identities with PtHY5, GhHY5, and AtHY5 (Fig. 1b−d). The predicting 3D structure of BpHY5 was shown in Fig. 1e. Analysis of cis-acting elements showed the promoter of *BpHY5* contained numerous stress-responsive elements (such as ABRE, ARE), light-responsive elements and hormone-responsive elements (such as abscisic acid) (Fig. 1f). Further, BpHY5-GFP signal distribution was strictly restricted to the nucleus, supporting the prediction that BpHY5 acted as a TF in the nucleus (Fig. 1g). The results indicated that BpHY5 belonged to bZIP family and could be involved in regulating stress responses.

### Expression profiles of *BpHY5*

**Figure 2:**
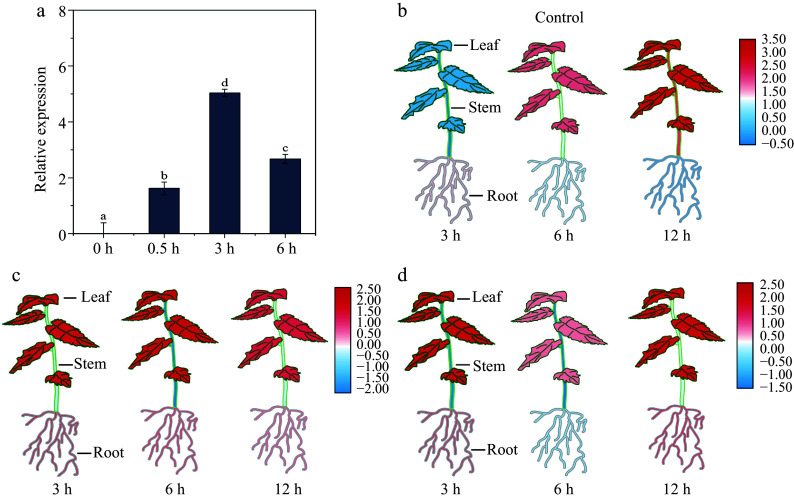
The expression profiles of *BpHY5*. (a) The expression level of *BpHY5* after 0, 0.5, 3, and 6 h treatment of 0.3 W/m^2^ UV-B. Heatmap of *BpHY5* expression under (b) control, (c) Mannitol, and (d) ABA treatments. Significance was determined by Student's t-test (*p* < 0.05).

To study the potential roles of *BpHY5*, we employed qRT-PCR to assess the expression levels of *BpHY5* in various treatments. It showed that the expression reached a peak at 3 h under 0.3 W/m^2^ UV-B (Fig. 2a). The expression levels of *BpHY5* in roots induced by mannitol peaked at 12 h (2.10-fold), while the expression levels in stems and leaves peaked at 3 h (1.42-fold and 4.05-fold) (Fig. 2c). The upregulation of *BpHY5* induced by ABA occurred at 12 h in roots (3.05-fold) and 3 h in leaves (4.57-fold) (Fig. 2d). These results indicated that the expression levels of *BpHY5* were induced by UV-B, Mannitol, and ABA, suggesting *BpHY5* was involved in responses to abiotic stresses, which was consistent with the analysis of the promoter cis-acting elements.

Furthermore, the expression level of *BpHY5* at different time points in one day (16 h light/8 h dark) showed that *BpHY5* was induced by light (Supplemental Fig. S1a). The *hy5* displayed drastically elongated hypocotyls and *BpHY5/*Col displayed shortened hypocotyls in both light and darkness, whereas the hypocotyls length of *BpHY5/hy5* was similar to Col, which indicated *BpHY5* restored *hy5* mutant phenotype and had similar functions to *AtHY5* (Supplemental Fig. S1b, c).

### Overexpression of *BpHY5* increases the UV-B tolerance

**Figure 3:**
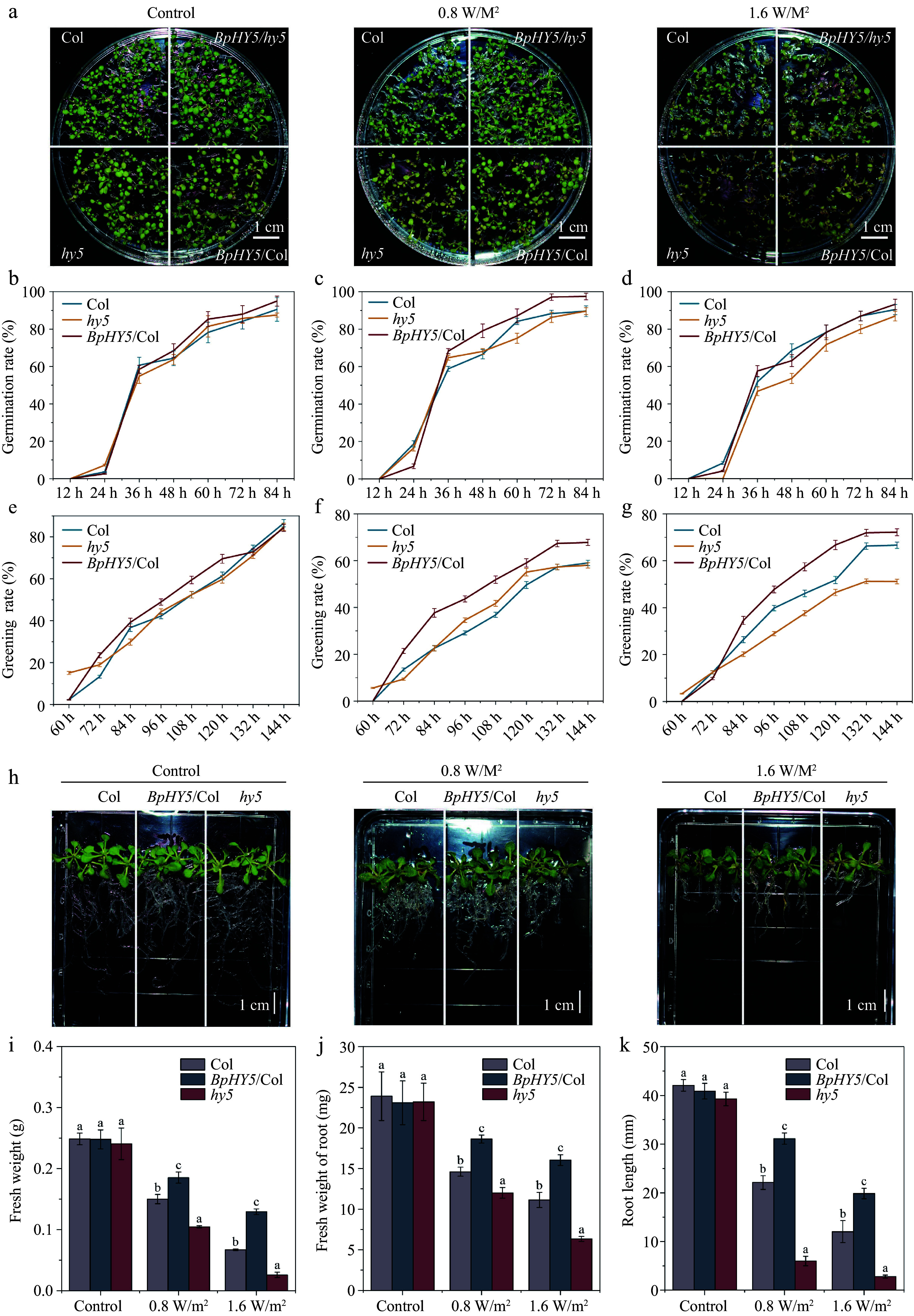
Comparison of germination, greening rate, root length, and fresh weight between *BpHY5*/Col, *hy5,* and Col under UV-B treatment. (a) Physical phenotypes of Col, *hy5*, *BpHY5*/*hy5,* and *BpHY5*/Col lines under 0.8 and 1.6 W/m^2^ UV-B treatment after 6 d. The germination rate of Col, *BpHY5*/Col, and *hy5* under (b) control, (c) 0.8, and (d) 1.6 W/m^2^ UV-B. The greening rate of Col, *BpHY5*/Col, and *hy5* under (e) control, (f) 0.8, and (g) 1.6 W/m^2^ UV-B. (h) The phenotypes of Col, *BpHY5*/Col, and *hy5* seedlings exposed to UV-B at 14 d. The statistics of (i) fresh weight, (j) fresh weight of root, and (k) root length of Col, *BpHY5*/Col, and *hy5* under 0.8 and 1.6 W/m^2^ UV-B at 14 d. The scale bar represents 1 cm. Statistical significance (*p* < 0.05) between Col, *BpHY5*/Col, and *hy5* is indicated by a, b, and c.

To determine the effect of *BpHY5* on UV-B tolerance in transgenic *Arabidopsis*, the growth of plants exposed to UV-B was compared to normal growth. Although there was no discernible difference between Col, *hy5*, and *BpHY5*/Col under control, the germination rate of Col was lower than *BpHY5*/Col under 0.8 W/m^2^ UV-B and the greening rate of *BpHY5*/Col was higher than Col under both 0.8 and 1.6 W/m^2^ UV-B (Fig. 3a−g). The fresh weight of *BpHY5*/Col was significantly higher, which was 1.23-fold and 1.92-fold of Col under 0.8 and 1.6 W/m^2^ UV-B, whereas the fresh weight of Col was significantly higher than *hy5* (Fig. 3h−j). The root length of *BpHY5*/Col was increased 1.41-fold and 1.65-fold of Col under 0.8 and 1.6 W/m^2^ UV-B, respectively, whereas that of *hy5* was decreased by 72.82% and 76.79% compared to Col (Fig. 3k).

**Figure 4:**
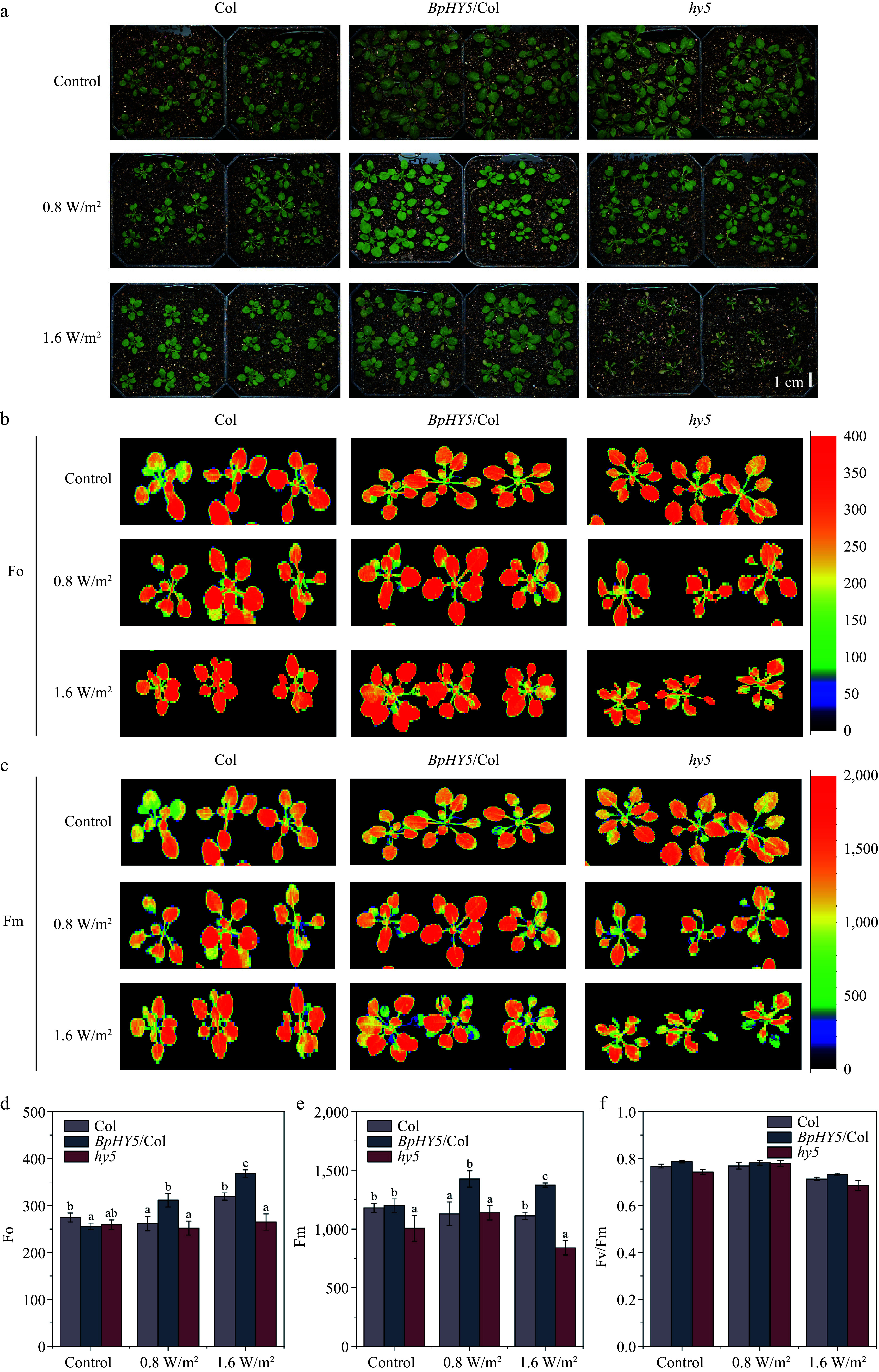
Transgenic *BpHY5* *Arabidopsis* showed higher UV-B tolerance. (a) Physical phenotypes of Col, *BpHY5*/Col, and *hy5* seedlings in soil under UV-B treatment. The chlorophyll fluorescence of Col, *BpHY5*/Col, and *hy5* seedlings after UV-B treatment for 10 d. Fo is (b) minimal fluorescence, and Fm is (c) maximal fluorescence. The statistics of (d) Fo, (e) Fm, and (f) Fv/Fm of Col, *BpHY5*/Col, and *hy5* under 0.8 and 1.6 W/m^2^ UV-B treatment after 10 d. The scale bar represents 1 cm. Significance was determined by Student's t-test (*p* < 0.05).

After 10 d of UV-B treatment, the size of *BpHY5*/Col was more developmental than Col and the *hy5* began to wilt and turned yellow and brown (Fig. 4a). The fresh weight of *BpHY5*/Col was 1.31-fold and 1.52-fold of Col under 0.8 and 1.6 W/m^2^ UV-B, respectively (Supplemental Fig. S2c). Under 0.8 and 1.6 W/m^2^ UV-B, Fo, and Fm of *BpHY5*/Col were significantly greater than those of Col, while Fo of *hy5* was lower (Fig. 4b−e). Furthermore, compared to Col, Fv/Fm of *BpHY5*/Col was higher under both control (1.03-fold of Col) and UV-B (1.02-fold and 1.03-fold of Col) conditions, indicating that overexpression of *BpHY5* promoted photosynthetic potential and increased the plant tolerance to UV-B (Fig. 4f). In conclusion, these results suggested that *BpHY5* positively regulated the plant resistance to UV-B.

### *BpHY5* improves the ROS scavenging ability and affects the cell wall components of stems under UV-B stress

**Figure 5:**
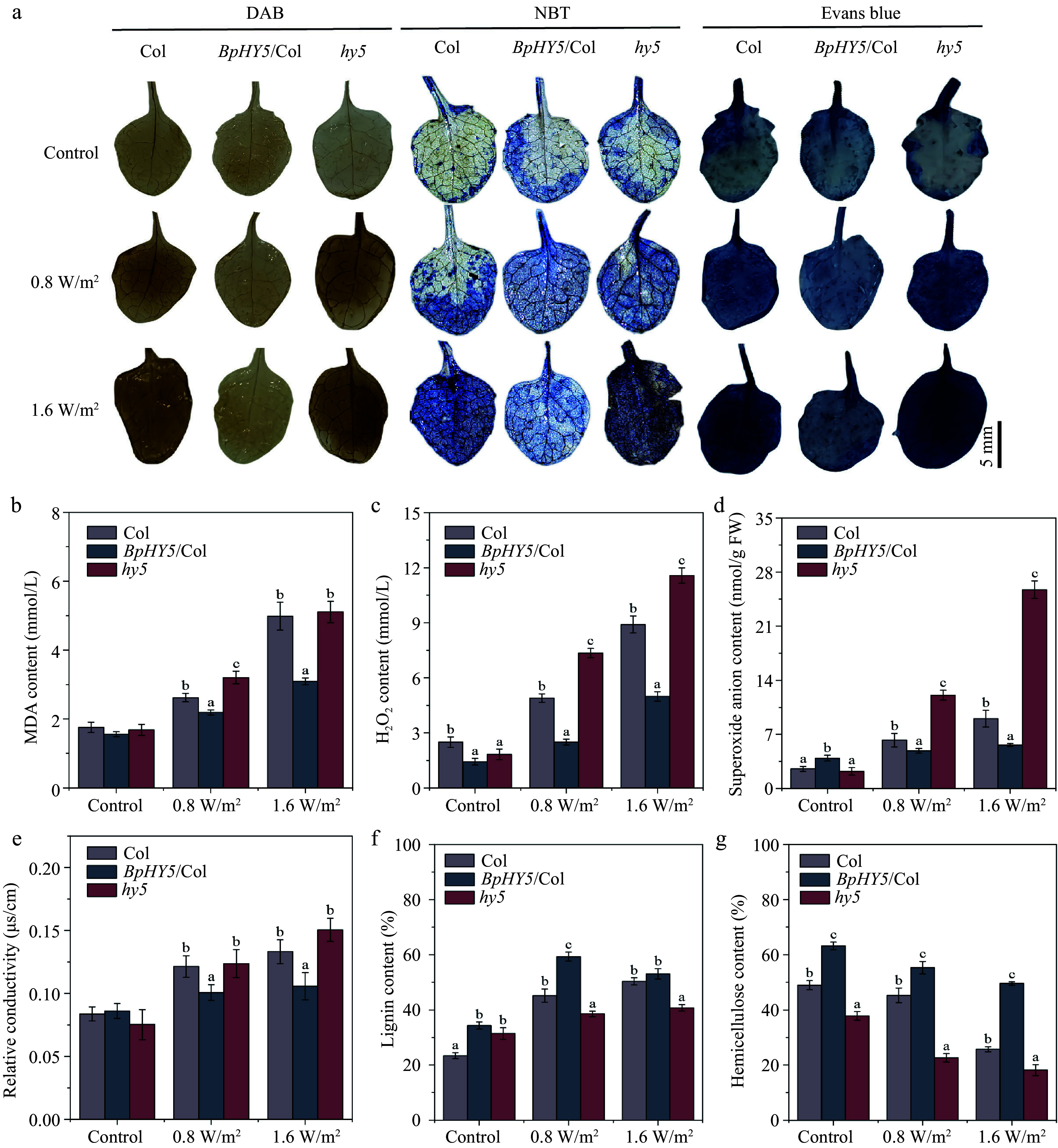
*BpHY5* affected the ROS accumulation and component content of stems under UV-B. (a) Determination of ROS content using DAB, NBT, and Evans blue staining. Scale bar represents 5 mm. The statistics of (b) MDA content, (c) hydrogen peroxide content, (d) superoxide anion content, and (e) relative conductivity. The statistics of component content of stems of Col, *BpHY5*/Col, and *hy5* after 10 d of treatment of 0.8 and 1.6 W/m^2^ UV-B, including (f) lignin content and (g) hemicellulose content. Significance was determined by Student's t-test (*p* < 0.05).

After 10 d treatment with UV-B, *BpHY5*/Col exhibited less damage compared to *hy5* and Col. The *BpHY5*/Col had lower brown coloration or blue spots than Col and *hy5* (Fig. 5a). The MDA, H_2_O_2_, superoxide anion content, and relative conductivity of all *Arabidopsis* lines increased after UV-B treatment (Fig. 5b−e). As expected, compared to Col, *BpHY5*/Col had lower MDA content (0.83-fold, 0.62-fold), lower H_2_O_2_ content (0.51-fold, 0.56-fold), lower superoxide anion content (0.78-fold, 0.62-fold), and lower relative conductivity (0.83-fold, 0.79-fold). These findings demonstrated overexpressing *BpHY5* enhanced tolerance to UV-B radiation by inhibiting ROS accumulation.

Interestingly, under UV-B condition, the lignin content of *BpHY5*/Col was higher than that of Col (1.31-fold, 1.05-fold), whereas the lignin content of *hy5* was lower than that of Col (0.85-fold, 0.81-fold) (Fig. 5f). Similarly, the hemicellulose content in *BpHY5*/Col was approximately 22.26% and 92.77% higher than that in Col, whereas the hemicellulose content in *hy5* was 50.00% and 29.52% lower than that in Col (Fig. 5g). It is worth noting that under the control condition, the cellulose content of *BpHY5*/Col was increased by 18.53% compared to Col. However, the cellulose content of *BpHY5*/Col was only 9.49% higher than that of Col after 0.8 W/m^2^ UV-B treatment, whereas it was 11.44% lower than that of Col after 1.6 W/m^2^ UV-B treatment (Supplemental Fig. S2d). These results demonstrated that *BpHY5* promoted the accumulation of lignin and hemicellulose.

### *BpHY5* is involved in the response to drought stress and ABA

**Figure 6:**
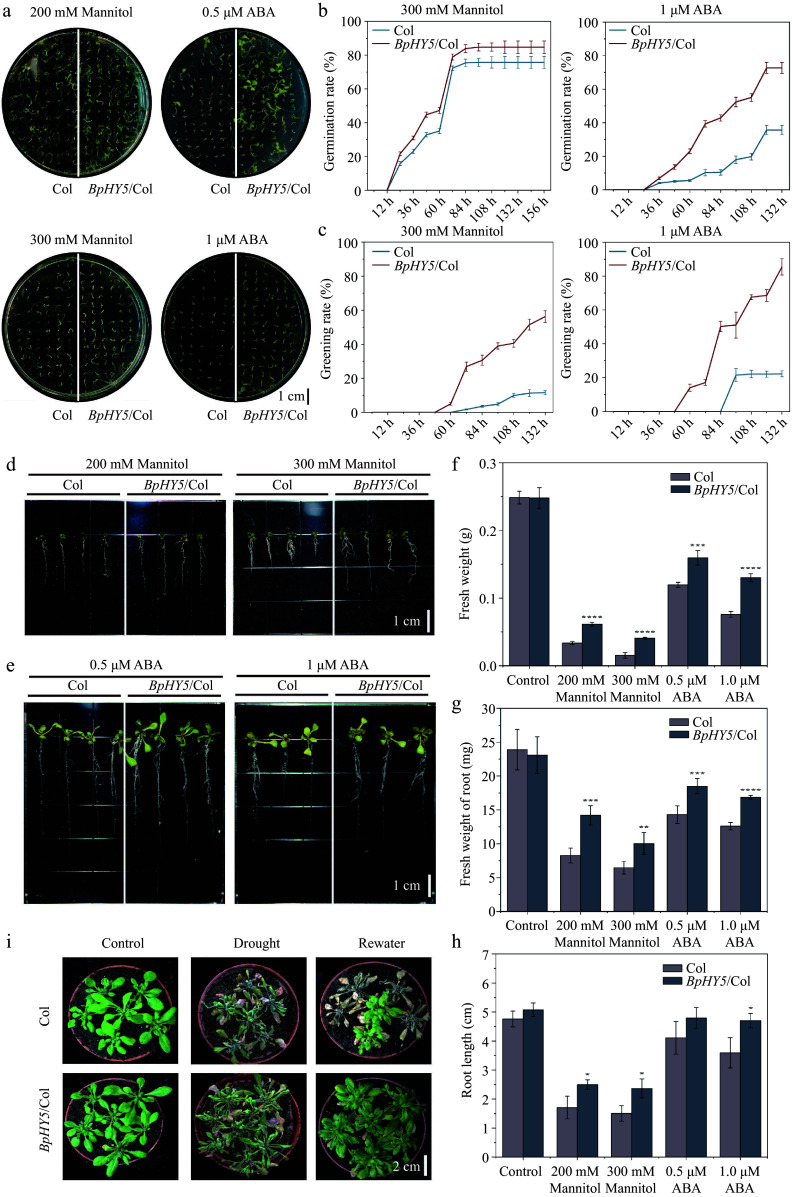
*BpHY5* decreased the sensitivity to drought and ABA in *Arabidopsis*. (a) Phenotypes of seed germination rate of Col and *BpHY5*/Col under 200, 300 mM mannitol and 0.5, 1.0 μM ABA at 7 d. Comparison of (b) germination rate and (c) greening rate of Col and *BpHY5*/Col under 300 mM mannitol and 1.0 μM ABA conditions. The root length phenotypes of Col and *BpHY5*/Col under (d) 200, 300 mM mannitol and (e) 0.5, 1.0 μM ABA treatments after 16 d. The statistics of the (f) fresh weight, (g) fresh weight of root, and (h) root length of Col and *BpHY5*/Col under mannitol (200, 300 mM) and ABA (0.5, 1.0 μM) treatment after 16 d. (i) Physical phenotypes of Col and *BpHY5*/Col subjected to drought stress without water for 7 d and re-watered for 3 d. Asterisks indicate levels of significance (t-test; *p < 0.05, **p < 0.01, ***p < 0.001, ****p < 0.0001). Scale bars: (a) = 1 cm; (d) = 1 cm; (e) = 1 cm; (i) = 2 cm.

The experiment exposed the seeds of *BpHY5*/Col and Col to different concentrations of mannitol and ABA to investigate the role of *BpHY5* in drought response. The exogenous ABA was added to model dehydration reactions. *BpHY5*/Col had significantly higher germination and greening rates than Col when treated with mannitol and ABA (Fig. 6a−c). After 16 d, the fresh weight of *BpHY5*/Col increased 1.83-fold and 2.64-fold of Col, and the root length of *BpHY5*/Col was 46.50% and 57.43% higher than Col under 200 mM and 300 mM mannitol, respectively. The fresh weight of *BpHY5*/Col roots was 1.29 times and 1.34 times as large as Col, and the root length of *BpHY5*/Col increased 1.17-fold and 1.31-fold of Col after 0.5 μM and 1 μM ABA treatment. In addition, the drought stress caused curly and wilted leaves in both *BpHY5*/Col and Col, but Col exhibited more damage than *BpHY5*/Col. After 3 d of rewatering, the withered leaves of *BpHY5*/Col recovered, whereas most of the leaves of Col did not recover and died (Fig. 6i). These results indicated that overexpression of *BpHY5* resulted in increased drought tolerance and reduced ABA sensitivity.

### Overexpression of *BpHY5* improves drought and ABA resistance in *Arabidopsis* and birch

**Figure 7:**
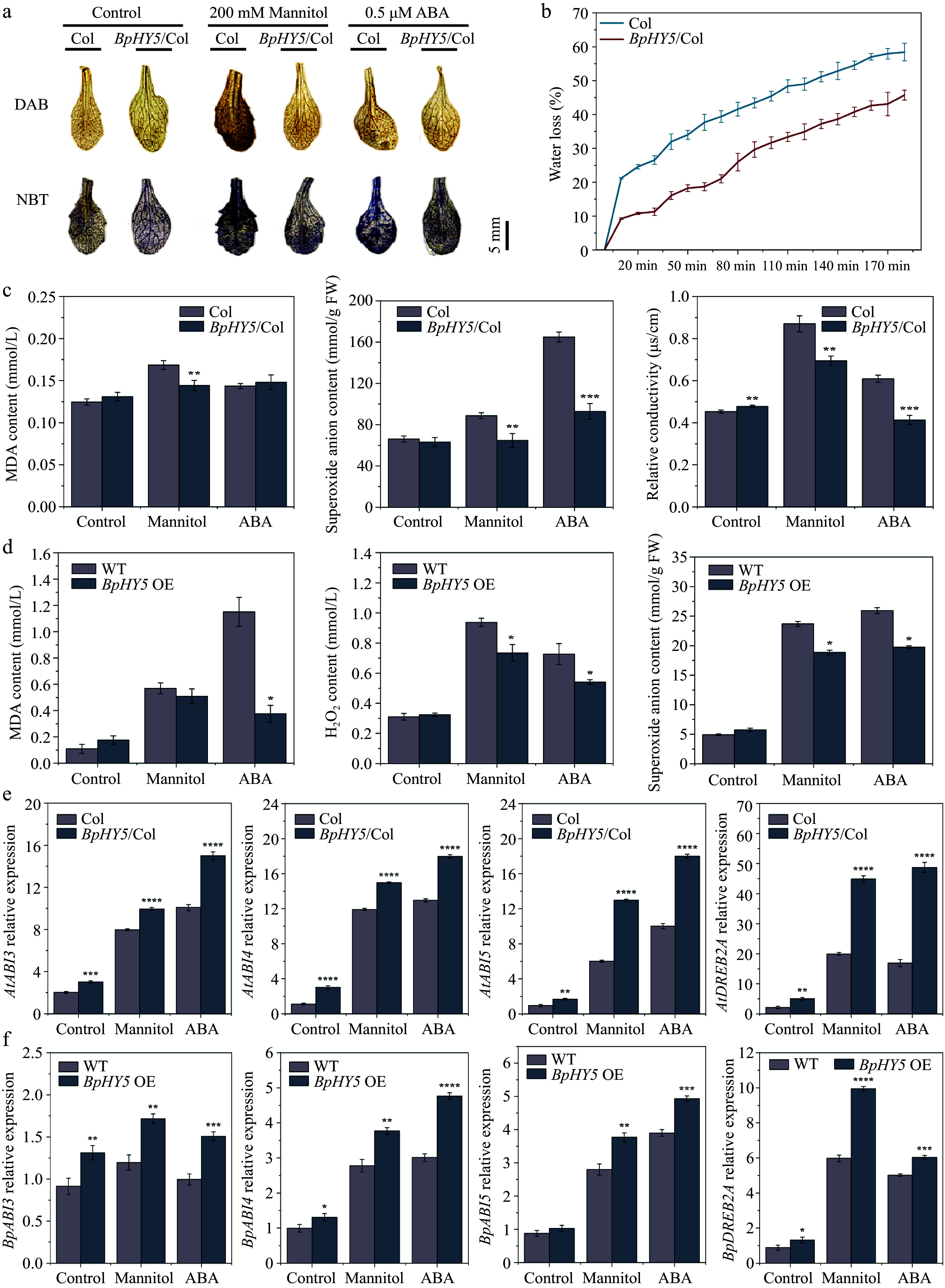
Effect of *BpHY5* overexpression on biochemical indicators and gene expression in *Arabidopsis* and birch under mannitol and ABA conditions. (a) DAB staining and NBT staining. Scale bar represents 5 mm. (b) Water loss rate of 16-day-old Col and *BpHY5*/Col seedlings. (c) The statistics of MDA content, superoxide anion content, and relative conductivity of 16-day-old Col and *BpHY5*/Col seedlings under mannitol and ABA conditions. (d) The statistics of MDA content, hydrogen peroxide content, and superoxide anion content of WT and transient transgenic birch under mannitol and ABA conditions. (e) The expression levels of *AtABI3*/*4*/*5* and *AtDEEB2A* in Col and *BpHY5*/Col under mannitol and ABA conditions. (f) The expression levels of *BpABI3*/*4*/*5* and *BpDEEB2A* in birch under mannitol and ABA conditions. Asterisks indicate levels of significance between Col and *BpHY5*/Col or WT and *BpHY5* OE (t-test; *p < 0.05, **p < 0.01, ***p < 0.001, ****p < 0.0001).

Plants exposed to mannitol and ABA were stained deeper by DAB and NBT than those under control conditions, suggesting that mannitol and ABA caused damage to plants (Fig. 7a). What stands out was that Col showed greater brown coloration and blue spots compared to *BpHY5*/Col under different stress conditions, demonstrating that the increased expression of *BpHY5* resulted in improved activity of scavenging ROS. Water loss rate, MDA, superoxide anion content, relative conductivity, and H_2_O_2_ are important evaluation indicators for plant damage. The water loss rate of *BpHY5*/Col was lower than that of Col during the period of dehydration (Fig. 7b). The MDA content, superoxide anion content, and relative conductivity of *BpHY5*/Col were all significantly lower than those of Col under mannitol and ABA treatments (Fig. 7c).

To further investigate the impact of *BpHY5* on drought and ABA resistance in birch, birch seedlings were transiently transformed and treated with mannitol and ABA. For accurately determining the degree of damage, MDA, H_2_O_2,_ and superoxide anion content were measured (Fig. 7d). There was a 10.53% and 67.36% decrease in MDA content of *BpHY5*/Col under mannitol and ABA treatments compared to Col, respectively. The H_2_O_2_ content of *BpHY5*/Col was 0.78-fold and 0.74-fold of Col under different stresses. Likewise, compared to Col, the superoxide anion content of *BpHY5*/Col decreased by 20.41% and 23.84% under different treatments. These results also demonstrated that *BpHY5* participated in the plant responses to drought and ABA with inhibited membrane damage, improved ROS scavenging ability, and enhanced tolerance to drought and ABA.

### Overexpression of *BpHY5* affects the expression of stress-related genes

**Figure 8:**
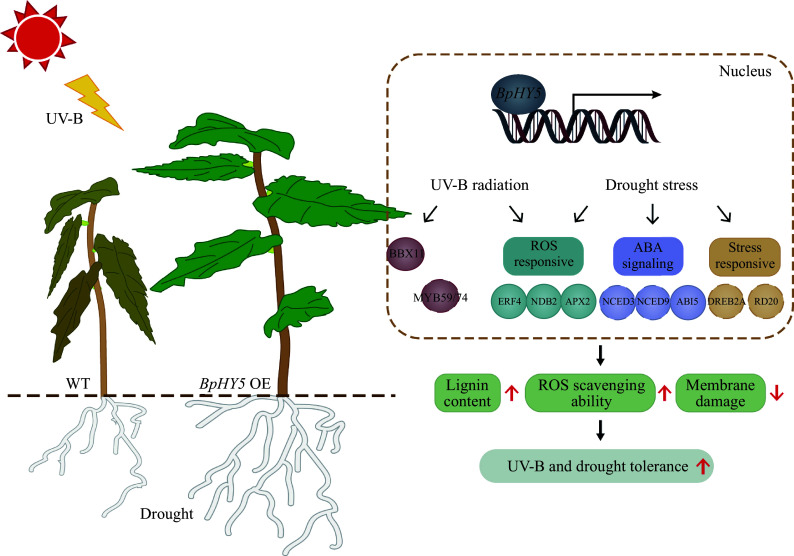
Schematic model of UV-B and drought stress response of *BpHY5*. Overexpression of *BpHY5* promotes the expression of *BBX* TFs, ABA-related genes (such as *NCED3/6/9* and *ABI3/4/5*), increases the expression of stress-response genes (such as *DREB2A*, *MYB59/74*, *ERD10* and *RD20*) and ROS-response genes (such as *ERF4*, *NDB2* and *APX2*) to enhance the plant tolerance to UV-B and drought stresses with higher lignin content, greater ROS scavenging ability and lower membrane damage. It has been demonstrated that HY5 binds the promoter of TFs in the solid line through previous studies; however, the TFs represented by dotted lines have not been explicitly demonstrated.

The expression of stress-related genes was evaluated to explore whether they could be induced by *BpHY5* (Supplemental Fig. S2g, h). The expression levels of some *B-box protein* (*BBX*) transcription factors related to UV-B response (such as *BBX4*, *BBX11*, *BBX21*, *BBX22*) were up-regulated in transgenic *Arabidopsis* and birch. The expression of ABA biosynthesis-related genes was up-regulated in *BpHY5* OE, for example, the expression levels of *BpNCED3* and *BpNCED9* in *BpHY5* OE were 15.08-fold and 18.41-fold of WT. Some genes responded to abiotic stresses and ABA, such as *MYB59*, *MYB74*, *ERD10,* and *RD20*, were up-regulated in *BpHY5*/Col and *BpHY5* OE. The transgenic plants showed significantly higher expression of ABA signaling pathway genes, including *AtABI3/4/5*, *BpABI3/4/5*, *AtDREB2A*, and *BpDREB2A* (Fig. 7e, f). Surprisingly, compared to control, the expression of *BpDREB2A* in birch overexpressing *BpHY5* increased 7.54-fold and 4.57-fold under mannitol and ABA treatments. Furthermore, the expression of ROS signaling-related genes (such as *ERF4*, *NDB2*, and *APX2*) was up-regulated in transgenic *Arabidopsis* and birch. In particular, the expression level of *BpERF4* in *BpHY5* OE was 15.56-fold of WT. In summary, these results showed that overexpression of *BpHY5* improved the expression of ABA signaling, ROS-responsive, and stress-responsive genes, promoted ABA signal transduction and enhanced the tolerance to UV-B and drought stress (Fig. 8).

## Discussion

HY5 is a member of bZIP TFs, which is involved in seed maturation, plant growth and development, light signal transduction, biosynthesis of plant secondary metabolites, and stress responses. In the present study, we cloned *BpHY5* in birch and explored the functions of *BpHY5* in response to UV-B radiation and drought stress.

### BpHY5 enhances plant UV-B tolerance which may be related to the higher lignin content

UV-B radiation acts as a cue for photomorphogenesis as well as a stress factor, depending on the dose^[15]^. *Arabidopsis* has two pathways for responding to UV-B radiation: a non-specific pathway (high level of UV-B causing DNA damage and ROS production) and a specific pathway (mediated by low level of radiation)^[16]^. The BBX11-HY5 feedback loop regulates tolerance under UV-B by promoting the accumulation of antioxidants and inducing the expression of photoprotection (ELIP, CHI, CHS) and DNA repair genes (UVR2, UVR3)^[17]^. In *Arabidopsis*, the promoter of *DEWAX* is bound directly and negatively regulated by HY5 to decrease plant sensitivity to UV-B^[6]^. The study has identified *BpUVR8* as a positive regulator in the photomorphogenesis mediated by UV-B in birch, positively regulating *BpHY5*^[13]^. According to our study, the expression level of *BpHY5* was induced by UV-B (Fig. 2a). Both 0.8 and 1.6 W/m^2^ UV-B decreased the germination and greening rate of seeds, reduced the root elongation, fresh weight, and photosynthetic potential, and increased the ROS activity leading to the damage of cell membrane, suggesting that high level of UV-B inhibited the plant growth and development (Figs 3−5). The germination rate and greening rate of *BpHY5*/Col was higher than Col under 0.8 W/m^2^ UV-B. The fresh weight and root length of *BpHY5*/Col was significantly higher than Col under the UV-B condition. The leaves and plant size of *BpHY5*/Col were much more developmental than Col under VU-B, whereas that of *hy5* was smaller than Col. Photosynthetic efficiency is affected by stress conditions. Fv/Fm is a parameter that reflects the health and growth by measuring plants' potential maximum photosynthetic capacity^[18]^. Fv/Fm of *BpHY5*/Col was higher under UV-B than Col, which indicated that overexpression of *BpHY5* promoted photosynthetic potential. The MDA, H_2_O_2_, superoxide anion content, and relative conductivity of plants overexpressed *BpHY5* was lower than Col, suggesting that *BpHY5* regulated the tolerance to UV-B by increasing the ROS scavenging activity and decreasing the damage degree of the cell membrane.

The plant cell wall is a dynamic structure that provides mechanical support and participates in stress responses by regulating cell wall biosynthesis^[19]^. Previous studies have shown that various abiotic stresses, such as drought and waterlogging, can significantly affect the cell wall composition, and structural rearrangement and change the lignin content^[20]^. UV-B radiation stimulates the phenylpropanoid pathway and the cotyledons of *Cucumis sativus* L. accumulate more lignin^[21]^. It is thought that lignin deposition in the epidermis of quinoa acts as a protective mechanism against UV-B rays^[22]^. The transgenic rice with increased lignin content shows higher UV-B resistance^[23]^. ChS2, a type of *Camellia sinensis* L., with higher resistance against UV-B radiation, shows a lignin-like layer on the callus surface. Exposure to UV-B radiation results in a notable increase in lignin accumulation, indicating that lignin plays a crucial role in cellular protection against UV-B^[24]^. To further explore the mechanism of *BpHY5* in regulating UV-B resistance, cell wall components were analyzed. In the present study, the lignin content of *BpHY5*/Col was higher than Col under control and UV-B conditions and the lignin content of *hy5* was lower than Col under UV-B stress, which was in line with previous results (Fig. 5f).

### BpHY5 functions as a stress-related transcription factor by regulating gene expression

Since the expression of over 3,000 genes can be directly or indirectly regulated by *HY5*, *HY5* responds to various stresses and hormonal signals^[25]^. In *Arabidopsis*, the CRYPTOCHROME2 (CRY2)-COP1-HY5-BBX7/8 module regulates cold acclimation mediated by blue light^[26]^. The coordinated action of *HY5* and *MYB15* positively regulates cold tolerance by controlling the expression of CBFs in tomatoes^[27]^. HY5 and HISTONE DEACETYLASE 9 (HDA9) are degraded by high temperature, which releases the binding to the promoter of *HSfA2* to promote gene expression and reduce salt tolerance^[28]^. However, previous research on responding to stresses to date has tended to focus more on roles in cold and salt tolerance than its effects on UV-B radiation and drought resistance.

In the present study, the BpHY5 protein was found to be located within the nucleus, suggesting that *BpHY5* acted as a TF (Fig. 1). And *BpHY5* might have regulated plant tolerance to abiotic stresses by controlling the expression of downstream genes. *NtHY5* affects the lignification of interfascicular and vascular tissues^[29]^. *MYB59* and *MYB74* with HY5 binding sites are positively regulated by HY5 and associated with secondary cell wall synthesis^[5,30]^. In the present study, the expression levels of *MYB59* and *MYB74* were up-regulated in *BpHY5*/Col, which suggested that the strengthening of UV-B resistance mediated by *BpHY5* may be connected to the increased lignin content and improved expression of genes associated with cell wall components biosynthesis.

Furthermore, we also confirm the expression levels of *BBX* genes. *HY5* positively regulates the expression of *BBX4*/*11*/*21*/*22*, while negatively regulates *BBX30*/*31* at the expression levels^[31]^. In *Arabidopsis*, *BBX11* promotes the accumulation of antioxidants and induces the expression of DNA repair genes to protect plants from high levels of UV-B. HY5 binds to the promoter of *BBX11* and overexpressing *BBX11* has been found to alleviate the high sensitivity to UV-B radiation observed in *hy5* to a certain extent^[17]^. Besides*, BBX20/21/22* have been identified as rate-limiting cofactors of *HY5*^[32]^. In the current research, the expression of *AtBBX11*, *BpBBX4*, *BpBBX21,* and *BpBBX22* were up-regulated in transgenic *Arabidopsis* and birch, which was consistent with previous research results (Fig. 7). These results identified that *BpHY5* was a positive transcription factor of plant tolerance to UV-B stress by regulating related-genes expression, such as *MYB59*, *MYB74,* and *BBX11*.

### BpHY5 improves plant tolerance to drought which may be related to ABA biosynthesis and signal transduction

In tomatoes, *HY5* plays a key role in green light-induced drought responses^[33]^. In the present study, analysis of cis-acting elements showed the promoter of *BpHY5* contained stress response elements and hormone response elements (such as abscisic acid), in which ABRE was an ABA-response element and always involved in ABA signal transduction (Fig. 1f). To explore the gene function of *BpHY5* in drought stress, transgenic plants were treated by mannitol and ABA (exogenous ABA was added to model dehydration reactions). The germination and greening rates, fresh weight, and root length of *BpHY5*/Col were significantly higher than Col under mannitol and ABA-added conditions. The plants of *BpHY5*/Col recovered after 3 d of rewatering, whereas most of Col did not recover and died (Fig. 6). Furthermore, the MDA, superoxide anion, H_2_O_2_ content and relative conductivity of *BpHY5* OE were decreased compared to wild type under drought treatment (Fig. 7). These results suggested that overexpression of *BpHY5* enhanced the plant tolerance to drought and decreased sensitivity to ABA by increasing ROS scavenging activity and decreasing membrane damage.

According to the results of qRT-PCR, it was noted that there were some differentially expressed genes associated with ABA signaling. ABA plays a vital role in the adaptation of plants to environmental stresses, especially young seedlings. NCED is regarded as the rate-limiting step of ABA biosynthesis^[34]^. *NCED3* promotes ABA accumulation and cooperates with *NCED5* to protect against drought stress^[35,36]^. Previous research has shown that *AtNCED6* and *AtNCED9* are necessary for ABA biosynthesis during seed development^[37]^. Analysis of HY5 genomic binding sites indicated that *AtNCED3* and *AtNCED9* are genes positively regulated by *AtHY5*^[5]^. As expected, our data showed that *AtNCED6*, *AtNCED9*, *BpNCED3,* and *BpNCED9* were up-regulated in overexpression lines (Fig. 7). Especially, the expression of *BpNCED3* and *BpNCED9* in birch overexpressed was 15.08-fold and 18.41-fold of WT, respectively.

*ABI3/4/5* have functions that overlap in ABA signaling transduction. *ABI5* is the crucial positive regulator in ABA signal. *HvABI5* regulates the drought response in an ABA-dependent manner^[38]^. Transgenic cotton expressing *AtABI5* exhibited higher resistance to drought and increased photosynthesis with greater root system and leaf area^[39]^. *HY5* and *ABI5* physically interact in response to ABA signaling in *Arabidopsis*, and *HY5* positively regulates ABA signaling^[40]^. The studies proved that HY5 binds to the promoter of *ABI5* to initiate the expression of *ABI5* and ABI5-targeting genes^[7,41]^. The present findings indicate that *BpHY5* overexpression resulted in a significant increase in *AtABI3/4/5,* and *BpABI3/4/5* expression levels than WT plants under control, drought, and ABA conditions, and the expression of *AtABI5* (7.71-fold) and *BpABI5* (3.70-fold) was induced by drought (Fig. 7). These findings indicated the enhancement of drought resistance mediated by *BpHY5* may be connected to the expression increase of genes associated to ABA, including *NCED3*, *NCED9,* and *ABI5*.

*DREB2A* and *DREB2B* are hypothesized to be the main TFs activated by drought stress. Overexpression of *DREB2A* significantly improves drought resistance in *Arabidopsis* and enhances drought, heat, and salinity tolerance in *Pennisetum glaucum*^[42,43]^. *RD20* is an ABA-responsive gene and participates in drought tolerance mechanisms by regulating stomatal aperture and plant growth^[44]^. The analysis results showed that *DREB2A* and *RD20* have the binding sites of HY5, and they are predicted to be the target genes of HY5^[5]^. However, there is no direct evidence of an interaction at present. Furthermore, previous studies document that HY5 binds to the promoter of ROS-responsive genes^[45]^. *ETHYLENE-RESPONSIVE TRANSCRIPTION FACTOR4* (*ERF4*), *NAD(P)H dehydrogenase B2* (*NDB2*), *Ascorbateperoxidase2* (*APX2*), belonging to ROS signaling pathways, are confirmed to be the key genes targeted by *HY5*^[45]^. The results of Sweetman et al. also suggest *HY5* mediated the transcription of *NDB2*^[46]^. The expression level of *APX2* is downregulated in the *hy5* mutant, which further proves that *APX2* is the downstream target gene of HY5^[47]^. Our results showed that the expression of the genes mentioned was up-regulated in transgenic *Arabidopsis* and birch. Especially, the expression level of *ERF4* in transgenic birch was 15.56-fold of WT (Fig. 7). The above results suggested that *BpHY5* enhanced drought tolerance by increasing transcript expression of stress- and ROS-responsive genes, including *DREB2A*, *RD20*, *ERF4*, *NDB2,* and *APX2*. However, we are also aware of several limitations of this study and the molecular mechanisms of *BpHY5* in UV-B and drought responses need to be further studied.

## Conclusions

In summary, *BpHY5* is a transcription factor of the bZIP family, which was highly induced by UV-B, drought, and ABA in the nucleus. Overexpression of *BpHY5* increased UV-B and drought tolerance and decreased ABA sensitivity, with longer roots, stronger ROS scavenging ability, lower damage degree, and higher stress-related gene expression levels. Overall, the present study indicated that *BpHY5* plays an important role in the plant stress endurance process, which suggests *BpHY5* is a promising candidate gene for birch breeding and provides a basis for further investigation of *BpHY5* functions in plant tolerance.

## SUPPLEMENTARY DATA

Supplementary data to this article can be found online.

## Data Availability

All data generated or analyzed during this study are included in this published article and its supplementary information files.

## References

[1] (2021). Differential responses of the scavenging systems for reactive oxygen species (ROS) and reactive carbonyl species (RCS) to UV-B irradiation in Arabidopsis thaliana and its high altitude perennial relative Arabis alpina. Photochemical & Photobiological Sciences.

[2] (2011). Perception of UV-B by the *Arabidopsis* UVR8 protein. Science.

[3] (2015). Q&A: how do plants sense and respond to UV-B radiation. BMC Biology.

[4] (1997). The *Arabidopsis HY5* gene encodes a bZIP protein that regulates stimulus-induced development of root and hypocotyl. Genes & Development.

[5] (2007). Analysis of transcription factor HY5 genomic binding sites revealed its hierarchical role in light regulation of development. The Plant Cell.

[6] (2020). ELONGATED HYPOCOTYL5 negatively regulates *DECREASE WAX BIOSYNTHESIS* to increase survival during UV-B stress. Plant Physiology.

[7] (2021). JMJ17–WRKY40 and HY5–ABI5 modules regulate the expression of ABA-responsive genes in Arabidopsis. New Phytologist.

[8] (2022). Abiotic stress responses in plants. Nature Reviews Genetics.

[9] (2017). ABP9, a maize bZIP transcription factor, enhances tolerance to salt and drought in transgenic cotton. Planta.

[10] (2016). Feedback regulation of ABA signaling and biosynthesis by a bZIP transcription factor targets drought-resistance-related genes. Plant Physiology.

[11] (2023). Basic helix-loop-helix transcription factor *PxbHLH02* enhances drought tolerance in *Populus* (*Populus simonii* × *P. nigra*). Tree Physiology.

[12] (2021). A method for determining the cutting efficiency of the CRISPR/Cas system in birch and poplar. Forestry Research.

[13] (2018). Molecular cloning and functional analysis of a UV-B photoreceptor gene, BpUVR8 (UV Resistance Locus 8), from birch and its role in ABA response. Plant Science.

[14] (2019). Molecular cloning and functional analysis of 4-Coumarate: CoA ligase 4 (*4CL-like 1*) from *Fraxinus mandshurica* and its role in abiotic stress tolerance and cell wall synthesis. BMC Plant Biology.

[15] (2021). Perception and signaling of Ultraviolet-B radiation in plants. Annual Review of Plant Biology.

[16] (2009). Signal transduction in responses to UV-B radiation. Annual Review of Plant Biology.

[17] (2022). Transcription factors BBX11 and HY5 interdependently regulate the molecular and metabolic responses to UV-B. Plant Physiology.

[18] (2022). Overexpression of *MdVQ37* reduces drought tolerance by altering leaf anatomy and SA homeostasis in transgenic apple. Tree Physiology.

[19] (2020). Maize *WI5* encodes an endo-1,4-β-xylanase required for secondary cell wall synthesis and water transport in xylem. Journal of Integrative Plant Biology.

[20] (2019). Structural features and regulation of lignin deposited upon biotic and abiotic stresses. Current Opinion in Biotechnology.

[21] (2007). Continuous UV-B irradiation induces morphological changes and the accumulation of polyphenolic compounds on the surface of cucumber cotyledons. Journal of Radiation Research.

[22] (2004). Epidermal lignin deposition in quinoa cotyledons in response to UV-B radiation. Photochemistry and Photobiology.

[23] (2007). Overexpression of rice WRKY89 enhances ultraviolet B tolerance and disease resistance in rice plants. Plant Molecular Biology.

[24] (2003). Effect of ultraviolet (UV-B) radiation on the formation and localization of phenolic compounds in tea plant callus cultures. Russian Journal of Plant Physiology.

[25] (2020). Chimeric activators and repressors define HY5 activity and reveal a light-regulated feedback mechanism. The Plant Cell.

[26] (2021). The CRY2–COP1–HY5–BBX7/8 module regulates blue light-dependent cold acclimation in Arabidopsis. The Plant Cell.

[27] (2020). The HY5 and MYB15 transcription factors positively regulate cold tolerance in tomato via the CBF pathway. Plant, Cell & Environment.

[28] (2023). HY5-HDA9 orchestrates the transcription of *HsfA2* to modulate salt stress response in *Arabidopsis*. Journal of Integrative Plant Biology.

[29] (2024). Mutation in shoot-to-root mobile transcription factor, ELONGATED HYPOCOTYL 5, leads to low nicotine levels in tobacco. Journal of Hazardous Materials.

[30] (2015). An *Arabidopsis* gene regulatory network for secondary cell wall synthesis. Nature.

[31] (2021). HY5: a pivotal regulator of light-dependent development in higher plants. Nature.

[32] (2020). Identification of BBX proteins as rate-limiting cofactors of HY5. Nature Plants.

[33] (2021). A transcriptome analysis revealing the new insight of green light on tomato plant growth and drought stress tolerance. Frontiers in Plant Science.

[34] (2000). Ectopic expression of a tomato 9-*cis*-epoxycarotenoid dioxygenase gene causes over-production of abscisic acid. The Plant Journal.

[35] (2001). Regulation of drought tolerance by gene manipulation of 9-*cis*-epoxycarotenoid dioxygenase, a key enzyme in abscisic acid biosynthesis in *Arabidopsis*. The Plant Journal.

[36] (2012). Epoxycarotenoid cleavage by NCED5 fine-tunes ABA accumulation and affects seed dormancy and drought tolerance with other NCED family members. The Plant Journal.

[37] (2006). Functional analysis of Arabidopsis *NCED6* and *NCED9* genes indicates that ABA synthesized in the endosperm is involved in the induction of seed dormancy. The Plant Journal.

[38] (2020). Barley *ABI5* (*Abscisic Acid INSENSITIVE 5*) is involved in abscisic acid-dependent drought response. Frontiers in Plant Science.

[39] (2014). Related to ABA-Insensitive3(ABI 3)/Viviparous1 and AtABI 5 transcription factor coexpression in cotton enhances drought stress adaptation. Plant Biotechnology Journal.

[40] (2021). HY5 and ABI5 transcription factors physically interact to fine tune light and ABA signaling in Arabidopsis. Plant Molecular Biology.

[41] (2008). Integration of light and abscisic acid signaling during seed germination and early seedling development. Proceedings of the National Academy of Sciences of the United States of America.

[42] (2006). Functional analysis of an *Arabidopsis* transcription factor, DREB2A, involved in drought-responsive gene expression. The Plant Cell.

[43] (2022). Expression of a *Pennisetum glaucum* gene *DREB2A* confers enhanced heat, drought and salinity tolerance in transgenic *Arabidopsis*. Molecular Biology Reports.

[44] (2010). RD20, a stress-inducible caleosin, participates in stomatal control, transpiration and drought tolerance in *Arabidopsis thaliana*. Plant and Cell Physiology.

[45] (2013). Antagonistic basic helix-loop-helix/bzip transcription factors form transcriptional modules that integrate light and reactive oxygen species signaling in *Arabidopsis*. The Plant Cell.

[46] (2022). Altering the balance between AOX1A and NDB2 expression affects a common set of transcripts in Arabidopsis. Frontiers in Plant Science.

[47] (2021). Arabidopsis downy mildew effector HaRxLL470 suppresses plant immunity by attenuating the DNA-binding activity of bZIP transcription factor HY5. New Phytologist.

