# Supplementary material for: A birch ELONGATED HYPOCOTYL 5 gene enhances UV-B and drought tolerance
Source: For Res (Fayettev). 2024 Jun 19;4:e022. doi: 10.48130/forres-0024-0019 (PMC11524257; doi:10.48130/forres-0024-0019)
Supplement: Supplementary file 1 — Supplementary data to this article can be found online. [file forres-0024-0019-S1.zip › 10.48130_forres-0024-0019-Suppl-FigureS2.pdf]

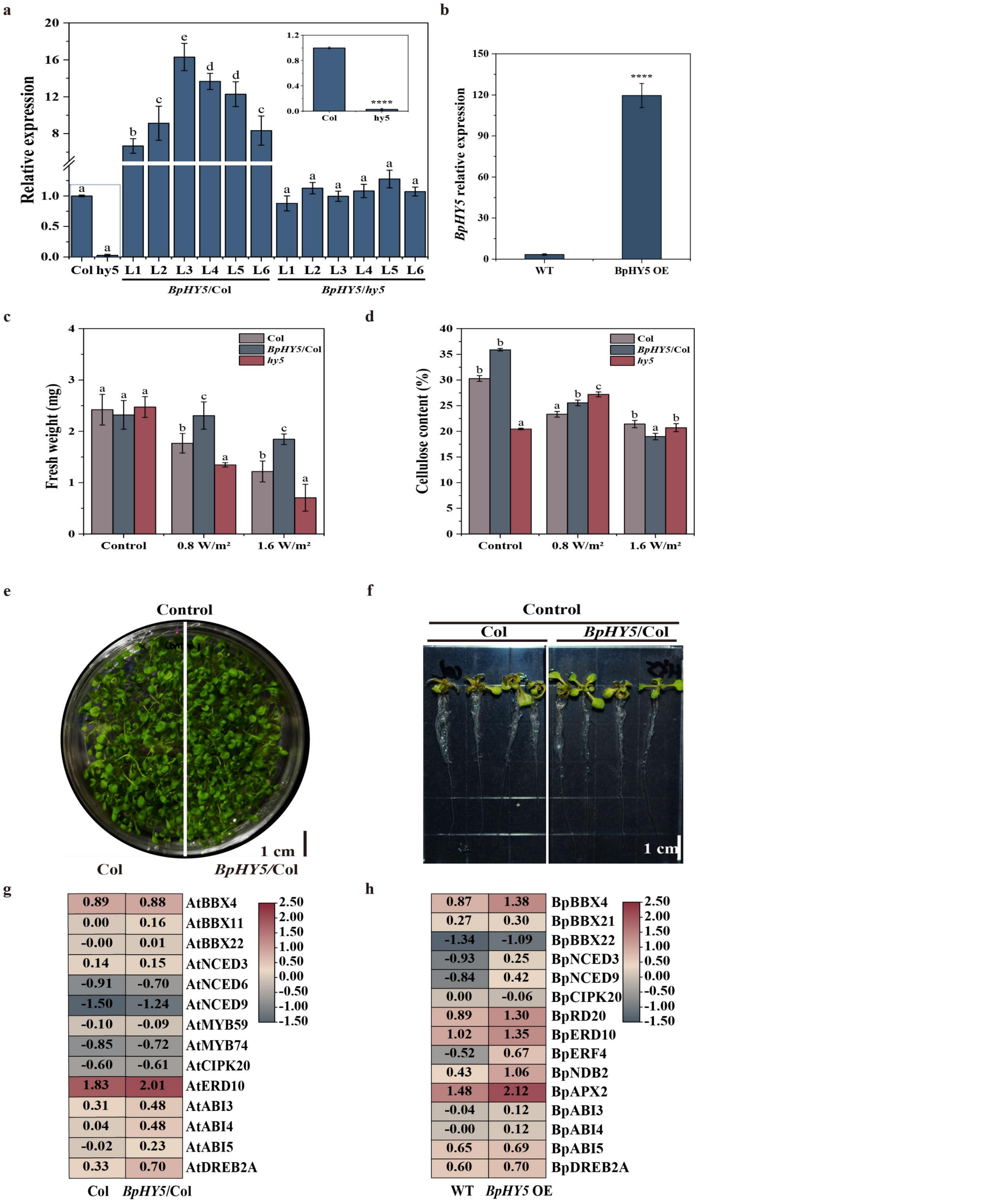

Fig. S2 (a) Expression levels of *BpHY5* in different transgenic lines of *Arabidopsis*. Statistical significance (p < 0.05) is indicated by a, b, c, d, e. (b) The relative expression level of *BpHY5* in transient transgenic birch and wild type. Data were represented as the mean ± SE of three biological replicates; asterisks indicate levels of significance (t-test; ****p < 0.0001). (c) The statistics of fresh weight of Col, *BpHY5*/Col, and *hy5* after 10 days of treatment of 0.8 W/m^2 and 1.6 W/m^2 UV-B. (d) The statistics of cellulose content of stems of Col, *BpHY5*/Col, and *hy5* after 10 days of treatment of 0.8 W/m^2 and 1.6 W/m^2 UV-B. Data are represented as the mean ± SE of three biological replicates; statistical significance ($p < 0.05$) between Col, *BpHY5*/Col, and *hy5* is indicated by a, b, c. (e) Phenotypes of seed germination rate of Col and *BpHY5*/Col under control condition at 7 d. (f) Phenotypes of root length of Col and *BpHY5*/Col under control condition at 16 d. (g, h) Heatmap of stress-related genes expression of control, transgenic *Arabidopsis* (g), and transient transgenic birch (h).
