# Supplementary material for: A birch ELONGATED HYPOCOTYL 5 gene enhances UV-B and drought tolerance
Source: For Res (Fayettev). 2024 Jun 19;4:e022. doi: 10.48130/forres-0024-0019 (PMC11524257; doi:10.48130/forres-0024-0019)
Supplement: Supplementary file 1 — Supplementary data to this article can be found online. [file forres-0024-0019-S1.zip › 10.48130_forres-0024-0019-Suppl-TableS1.pdf]

Table S1: Primer sequences

Primers for cloning	Sequence (5' to 3')
BpHY5-F	ATGCAAGAGCAGGCAACAAG
BpHY5-R	TTAAGACCCATCATTGCCACTAGC
**Primers for vector construction**	**Sequence (5' to 3')**
pROKII-BpHY5-GFP-F	ccccgggtaccgatactcgagATGCAAGAGCAGGCAACAAG
pROKII-BpHY5-GFP-R	agaaccacgggtcatctcgagAGACCCATCATTGCCACTAGC
pEarleyGate 103-35S -BpHY5-GFP-F	gcgtcgacATGCAAGAGCAGGCAACAAG
pEarleyGate 103-35S -BpHY5-GFP-R	ttgcggccgcAGACCCATCATTGCCACTAGC
**Primers for qRT-PCR**	**Sequence (5' to 3')**
At18S-F	GGAAAGGATCTGTACGGTAAC
At18S-R	TGTGAACGATTCCTGGAC
AtBBX4-F	CCACTCAGCTAATCCACTCTC
AtBBX4-R	GTCACCGCCATCTTCATCTAC
AtBBX11-F	AATCTCCCTCTTCCCTTTTCTG
AtBBX11-R	TGACTCCCAAAGCAATCCG
AtBBX22-F	AATAACTGCTTGGGAGGTGAG
AtBBX22-R	CTTAGGCCAGTTTAGACCAGAC
AtNCED3-F	CCAGCAAGTCGTTTTCAAGC
AtNCED3-R	TCATCTGTTTCTGGCTCTTCC
AtNCED6-F	TATGGAATGCGTGGGAAGAG
AtNCED6-R	ACCCGCTTCTAAATTCACTCC
AtNCED9-F	CAATACCAAAACCCCAAACCG
AtNCED9-R	GGTCTCGAAGAGGAAGATGAAG
AtMYB59-F	AGAGCGTTTAGTCCTTGAGC
AtMYB59-R	CTCATTATCTGTTCTCCCCGG
AtMYB74-F	ATACTCAAACTCGCAACCTCTC
AtMYB74-R	GCATGAGGTCTTGGAAATTCG
AtCIPK20-F	TGATGGAGCTAAAGCCGATG

AtCIPK20-R	GGTTAGGGTCAAGAATCCGAG
AtERD10-F	TCTGAACCAGAGTCGTTTGTG
AtERD10-R	CTTCTCACCGTCTTCACCTTC
AtABI3-F	GAAACTGTGACGACTCTTCTGGTG
AtABI3-R	GTGTCAAAGAACTCGTTGCTATCA
AtABI4-F	GCTTCCCAACATCAACACAACC
AtABI4-R	TCTCGGCGACCCATTTGC
AtABI5-F	ATGGTAACTAGAGAAACGAAG
AtABI5-R	GAAATCCTCAAGTGTCATCTC
AtDREB2A-F	AAGGTAAAGGAGGACCAGAG
AtDREB2A-R	ACACAACCAGGAGTCTCAAC
BpTUB-F	TCAACCGCCTTGTCTCTCAGG
BpTUB-R	TGGCTCGAATGCACTGTTGG
BpHY5-F	AGGTTGCTGAGGAATAGAGTGTC
BpHY5-R	TGCTGTTGTGTTCTTCAATAGG
BpBBX4-F	ACCGATGTGTCCGATTCTTAC
BpBBX4-R	GCGAACCTGCCTTTGATTC
BpBBX21-F	CAACAGCAATCACCGACAAG
BpBBX21-R	AGATCAGCATCCAGAAACGG
BpBBX22-F	GAAGATACAGTGCAACGTGTG
BpBBX22-R	GAAGTACCCAACTGACTCCTG
BpNCED3-F	GGTCGTAGTGATTGGCTCTTG
BpNCED3-R	GCTCAGAATCGGGTAAGATGG
BpNCED9-F	TTACCCGATTCTGAGCAAGTG
BpNCED9-R	CCAGTGAAGAGGTCTACTTTCG
BpCIPK20-F	AGGTTTTGAGGGTGGGATTG
BpCIPK20-R	TCTTCCTTGAGCTTCCCTTTG
BpRD20-F	TGGCGATCTTCCTGTTCATG
BpRD20-R	TGGCTCTCATCGTAGGAAATG
BpERD10-F	AATATCAAAGGACGGGCTGG

BpERD10-R	AGTTCTCCCCTTTGTTTCGG
BpERF4-F	AGAAAGAGGTGCATTACAGGG
BpERF4-R	TTTGGCAGAGGGAAGTTGG
BpNDB2-F	ATATGGAATGGCTGTCTGGTC
BpNDB2-R	CCAAGTGCATATACATTGTCGC
BpAPX2-F	CATTGTTGCTCTTTCTGGTGC
BpAPX2-R	AAAGATGAGAGGGTTGGAAGTC
BpABI3-F	ATGGTTCACTCTTTTACACCGACTT
BpABI3-R	CCATATCTTCCTCTGTGTCCGAC
BpABI4-F	TCATCACCCTCCCCAAGACAC
BpABI4-R	ACCTGAACTTGCTGTTGTCGG
BpABI5-F	AGAATCCCGAGTCTGCCCCT
BpABI5-R	CCCACCATAACAAACCCCCA
BpDREB2A-F	CTATGTCCGTGGCTGAAACCC
BpDREB2A-R	CTCTGGTCCTCCCTTCCCTTT
