# Supplementary material for: A birch ELONGATED HYPOCOTYL 5 gene enhances UV-B and drought tolerance
Source: For Res (Fayettev). 2024 Jun 19;4:e022. doi: 10.48130/forres-0024-0019 (PMC11524257; doi:10.48130/forres-0024-0019)
Supplement: Supplementary file 1 — Supplementary data to this article can be found online. [file forres-0024-0019-S1.zip › 10.48130_forres-0024-0019-Suppl-FigureS1.pdf]

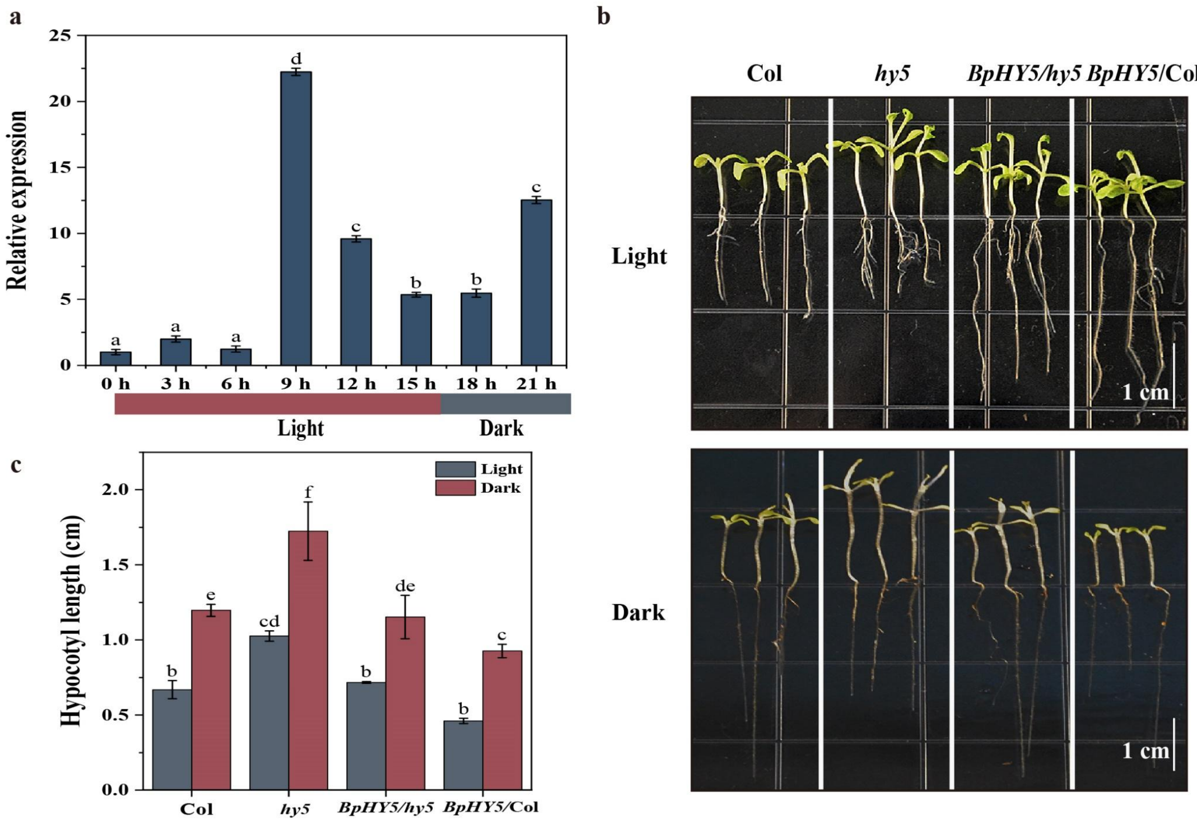

Fig. S1 (a) The expression level of *BpHY5* at 0, 3, 6, 9, 12, 15, 18 and 21 h in one day. (b) Phenotypes of seedlings of Col, *hy5*, *BpHY5*/*hy5,* and *BpHY5*/Col under visible light or darkness for 7 d. Scale bar represents 1 cm. (c) Hypocotyl length of Col, *hy5*, *BpHY5*/*hy5,* and *BpHY5*/Col under visible light or darkness for 7 d. Data are represented as the mean ± SE of three biological replicates; statistical significance (p < 0.05) is indicated by a, b, c, d, e, f.
